# Phase I–II study of docetaxel and ifosfamide combination in patients with anthracycline pretreated advanced breast cancer

**DOI:** 10.1038/sj.bjc.6600887

**Published:** 2003-04-15

**Authors:** C Kosmas, N Tsavaris, N Malamos, N Stavroyianni, A Gregoriou, S Rokana, A Polyzos

**Affiliations:** 1 Department of Medicine, Medical Oncology Unit, Helena-Venizelou Hospital, 21 Apolloniou Street, 16341, Athens, Greece; 2 Oncology Unit, Department of Pathophysiology, Athens University School of Medicine, Laikon General Hospital, Athens, Greece; 3 Oncology Unit, Department of Propedeutic Medicine, Athens University School of Medicine, Laikon General Hospital, Athens, Greece

**Keywords:** docetaxel, ifosfamide, breast cancer, phase I study

## Abstract

Given the established individual activity of docetaxel and ifosfamide in anthracycline pretreated advanced breast cancer, the present phase I–II study aimed to define the maximum tolerated dose (MTD), the dose-limiting toxicities (DLTs), and activity of the docetaxel–ifosfamide combination in this setting. Cohorts of three to six patients with histologically confirmed metastatic breast cancer after prior anthracycline-based chemotherapy were treated at successive dose levels (DLs) with escalated doses of docetaxel 70–100 mg m^−2^ over 1 h on day 1 followed by ifosfamide 5–6 g m^−2^ divided over days 1 and 2 (2.5–3.0 g m^−2^ day^−1^ over 1 h), and recycled every 21 days. G-CSF was added once dose-limiting neutropenia was encountered at a certain DL and planned to be incorporated prophylactically in subsequent higher DLs. In total, 56 patients with a median age of 54.5 (range, 32–72) years and performance status (WHO) of 1 (range, 0–2) were treated at five DLs as follows: 21 in phase I DLs (DL1: 3, DL2: 6, DL3: 3, DL4: 6, and DL5: 3) and the remaining 35 were treated at DL4 (total of 41 patients at DL4), which was defined as the level for phase II testing. All patients were assessable for toxicity and 53 for response. Dose-limiting toxicity (with the addition of G-CSF after DL2) was reached at DL5 with two out of three initial patients developing febrile neutropenia (FN). Clinical response rates, on an intention-to-treat basis, in phase II were: 53.6% (95% CI, 38.3–68.9%); three complete remissions, 19 partial remissions, seven stable disease, and 12 progressive disease. The median response duration was 7 months (3–24 months), median time to progression 6.5 month (0.1–26 month), and median overall survival 13 months (0.1–33 months). Grade 3/4 toxicities included time to progression neutropenia in 78% of patients–with 63% developing grade 4 neutropenia (⩽7 days) and in 12% of these FN, while no grade 3/4 thrombocytopenia was observed. Other toxicities included peripheral neuropathy grade 2 only in 12%, grade 1/2 reversible CNS toxicity in 17%, no renal toxicity, grade 2 myalgias in 10%, grade 3 diarrhoea in 10%, skin/nail toxicity in 17%, and grade 2 fluid retention in 2% of patients. One patient in the study treated at phase II died as a result of acute liver failure after the first cycle. In conclusion, the present phase I–II study determined the feasibility of the docetaxel–ifosfamide combination, defined the MTD and demonstrated the encouraging activity of the regimen in phase II, thus warranting further randomised phase III comparisons to single-agent docetaxel or combinations of the latter with other active agents.

Docetaxel represents a novel antimicrotubule agent that promotes the polymerisation of tubulin and thereof stabilises microtubules by preventing their disassembly. Docetaxel (Taxotere®) has demonstrated a broad spectrum of activity against a variety of advanced solid tumours, and breast cancer represents the first in which docetaxel has been successfully tested (Salminen *et al*, 1999). In particular, for patients with prior anthracycline-based therapy, taxanes represent the treatment of choice in the salvage setting, since previously applied agents have demonstrated inferior activity, taxanes have exhibited a relative lack of cross-resistance with anthracyclines, and have so far demonstrated fair tolerability in pretreated patients.

Three second-line phase III studies in anthracycline-refractory patients evaluated single-agent docetaxel *vs* salvage regimens thought to be active in this setting, namely mitomycin-C+vinblastine (Nabholtz *et al*, 1999), methotrexate–5-fluorouracil (5-FU) (Sjostrom *et al*, 1999), and infusional 5-FU+vinorelbine (Monnier *et al*, 1998). Two of the above studies (Nabholtz *et al*, 1999; Sjostrom *et al*, 1999) demonstrated an advantage in favour of docetaxel with respect to response rate (RR) and time to progression (TTP), while only the study of Nabholtz *et al* (1999) so far reported a significant 3-month prolongation in median overall survival (OS), while, in contrast, the third study by Monnier *et al* (1998) did not report any advantage of docetaxel *vs* infusional 5-FU+vinorelbine. Moreover, in a recently reported large phase III randomised trial (Chan *et al*, 1999) comparing single-agent docetaxel *vs* doxorubicin in alkylating agent pretreated metastatic breast cancer patients reported a significantly higher RR for docetaxel (52 *vs* 37%), without, however, prolongation in median TTP. The other taxane, paclitaxel, has been compared to doxorubicin in two recent large phase III studies (Sledge *et al*, 1997; Paridaens *et al*, 2000). The first study conducted by the EORTC yielded significantly higher RRs and longer progression-free survival in favour of doxorubicin (Paridaens *et al*, 2000). The second study, a three-arm North American trial, yielded equivalent results in terms of RR, TTP, and OS between the doxorubicin and paclitaxel single-agent arms (Sledge *et al*, 1997).

Ifosfamide, an oxazophosphorine alkylating agent like cyclophosphamide, has demonstrated substantial activity in advanced breast cancer (Ahmann *et al*, 1974). The rationale for combining docetaxel and ifosfamide derives from *in vitro* data indicating the ability of taxanes to revert the repair mechanisms responsible for the development of resistance to alkylating agent-induced DNA damage. A single previous phase I study has evaluated the feasibility of the docetaxel–ifosfamide combination without G-CSF in pretreated patients with a variety of advanced solid tumours. Dose-limiting toxicity (DLT) was reached at docetaxel 85 mg m^−2^ on day 1 followed by ifosfamide 5 g m^−2^ administered as 24-h infusion and the recommended phase II doses were docetaxel 75 mg m^−2^+ifosfamide 5 g m^−2^ (Pronk *et al*, 1998).

Given the encouraging activity of each individual cytotoxic agent and the feasibility of the docetaxel+ifosfamide combination, we elected to conduct a phase I/II study in an attempt to further intensify the above regimen, possibly with the aid of G-CSF, in patients with anthracycline-pretreated metastatic breast cancer and administer it in a total outpatient setting by avoiding the 24-h infusion and selecting the fractionated short-over 2 d-infusion of ifosfamide.

## PATIENTS AND METHODS

### Patient selection

Patients with histologically confirmed stage IV breast cancer pretreated with anthracycline-based chemotherapy were enrolled; patients progressing on anthracycline-based therapy or within 4 months after the end of such a treatment or patients treated with neoadjuvant and adjuvant anthracyclines that progressed within 12 months after the end of adjuvant chemotherapy were deemed anthracycline-refractory, while all other patients were considered potentially anthracycline-sensitive. Patients had to have bidimensionally measurable lesions with at least one outside a previously irradiated field, unless definite evidence of progression at this site was observed during a minimum 3-month period. No prior taxanes or ifosfamide were allowed. Other inclusion criteria were as follows: age 18–72 years; a World Health Organization (WHO) performance status (PS) of 0–2; life expectancy of at least 3 months; adequate haematopoietic (ANC⩾1500 *μ*l^−1^, PLT ⩾100 000 *μ*l^−1^), liver (bilirubin <1.5 mg dl^−1^, AST/ALT <2 × upper normal limit, unless caused by tumour and serum albumin >3.0 g dl^−1^) and renal function (BUN and creatinine <1.5 × upper normal limit; and creatinine clearance >50 ml min^−1^), and cardiac function (left ventricular ejection fraction (LVEF) ⩾50%). Patients with brain metastases were eligible provided that they had been irradiated and had clinical and radiological improvement and were off steroids or receiving tapering doses of steroids. Other exclusion criteria were radiation therapy within 4 weeks from treatment initiation, irradiation of more than 25% of the bone marrow-bearing skeleton, severe infection or malnutrition. The study was approved by the Ethical and Scientific Committees of the participating institutions and informed consent was obtained from each patient before study entry.

### Treatment schedule

Eligible patients were entered in the dose levels (DLs) as shown in Table 1

**Table 1:** Docetaxel–ifosfamide dose levels in the phase I part of the study

	**Drug doses**			
**Dose level**	**Docetaxel (mg m^−2^)**	**Ifosfamide (g m^−2^)**	**G-CSF**	**No. of patients entered**
1	70	5.0	−	3
2	85	5.0	−	6
3	85	5.0	+	3
4	100	5.0	+	6+35 (phase II)
5	100	6.0	+	3

. Docetaxel (Taxotere®) was administered at 70–100 mg m^−2^ over 1-h by i.v. infusion on day 1, after premedication consisting of dexamethasone 20 mg, dimethidene maleate (Fenistil®) 4 mg and ranitidine 50 mg; all administered i.v. 30 min before docetaxel. Ifosfamide followed docetaxel and was administered at 5.0–6.0 g m^−2^ divided over 2 days (2.5–3.0 g m^−2^ day^−1^ i.v. over 1 h) together with mesna uroprotection administered at 40% of the ifosfamide dose, together with ifosfamide within the same solution (1 l of 1/2 (0.9% normal saline+dextrose 5%)+20 meq KCl+30 meq bicarbonate and 10 mg furosemide), and 80% of the ifosfamide dose divided within the postifosfamide hydration fluids, which consisted of 2 l (1/2 (0.9% normal saline+dextrose 5%)+20 meq KCl+ 10 mg furosemide) administered over 6 h in the outpatient unit.

### Supportive care

Standard antiemetic medication included ondansetron 24 mg i.v. 1-h before chemotherapy on days 1, 2 and postchemotherapy 8 mg t.i.d. per os on days 3–5. Dexamethasone 20 mg i.v. was administered 1-h before chemotherapy (on day 1 as docetaxel premedication as well) on days 1, 2 and postchemotherapy 4 mg t.i.d. or methylprednisolone 16 mg b.i.d per os on days 3–5.

Haematopoietic growth factors included G-CSF (lenograstim) 150 *μ*g m^−2^ day^−1^ s.c. from day 4 until day 10 or until WBC⩾10000 *μ*l, whatever came first.

### Dose escalation schedule, DLTs, and dose modifications

Dose-limiting toxicities were assessed during the first chemotherapy cycle and were considered to have been reached when one of the following was met: (i) grade 4 neutropenia of >7 days duration, (ii) any episode of ⩾grade 3 febrile neutropenia, (iii) any episode of grade 4 thrombocytopenia, (iv) any nonhaematologic grade 3 or 4 toxicity excluding nausea/vomiting, musculoskeletal-arthritic pain, and alopecia.

Cohorts of three patients were entered at the dose levels shown in Table 1. In the case that DLT was encountered (defined above) in one out of three patients at a certain DL a total of six patients were entered at that particular level and if more than two out of six (33%) met the DLT requirements (in total at least three out of six patients developed the same DLT) no further accrual to the next higher DL was undertaken and the level immediately before the DLT was considered as the maximum tolerated dose (MTD). In the case that two out of the first three patients at a certain level experienced the same DLT, no more patients were accrued at that level and further dose escalation was stopped. The DL immediately before the one that DLT was reached, that is, the MTD, was recommended for further phase II testing.

The following guidelines were applied with respect to dose reductions for toxicity: (i) for neutropenia and thrombocytopenia, meeting the aforementioned criteria, docetaxel and ifosfamide doses were reduced by 20% in subsequent cycles and if toxicity reappeared after a total of 40% reduction from the starting dose in consecutive cycles, treatment was stopped; however, the patient was evaluable for toxicity and response, (ii) for ⩾grade 3 mucositis, the doses of docetaxel and ifosfamide were reduced by 20% in subsequent cycles, (iii) for neuropathy ⩾grade 3 treatment was interrupted, and (iv) for renal toxicity ⩾grade 3 toxicity (serum creatinine elevations>3 × normal) treatment was withheld until recovery (serum creatinine <1.8 mg dl^−1^) with ifosfamide administered with more posthydration and hospitalisation in subsequent cycles. If grade 3 or more renal toxicity persisted, ifosfamide was omitted in subsequent cycles. (v) For ⩾grade 3 CNS toxicity (ifosfamide encephalopathy), the dose of ifosfamide was reduced by 20% and more hydration with bicarbonates was anticipated in subsequent cycles. In the case that encephalopathy reappeared, then ifosfamide was omitted from subsequent cycles. In the case that blood counts had not recovered to ANC⩾1500 *μ*l^−1^ and PLT⩾100 000 *μ*l^−1^ on the day of therapy, treatment was withheld until recovery, and after a maximum delay of 2 weeks (day 35) no further therapy was administered in case that counts did not return to normal.

### Patient evaluation

Baseline evaluations included: patient history, physical examination, chest X-rays, complete blood count with differential and platelet count, blood chemistry (AST, ALT, sALP, gamma-GT, bilirubin (direct/indirect), protein, creatinine, BUN, uric acid, glucose, Na^+^, K^+^, Ca^2+^, P^−^), ECG, and echocardiography or multigated angiogram (MUGA) scan with LVEF measurement. Computed tomography (CT) scans of the chest, abdomen, pelvis, and bone scintigraphy were performed at study entry and CT scan of the brain whenever clinically indicated. Complete blood counts with differential and platelet counts were performed twice weekly or daily in case of grade 3/4 neutropenia, thrombocytopenia or febrile neutropenia until haematologic recovery; blood chemistry and physical examination were performed every 3 weeks. Toxicities were evaluated according to the NCI common toxicity criteria (NCI-CTC).

Responses (complete remission (CR), partial remission (PR), stable disease (SD), and progressive disease (PD)) were evaluated according to WHO response criteria (Miller *et al*, 1981).

Patients were evaluated before each cycle for lesions assessable by physical examination or chest X-ray; however, all patients were evaluated by the appropriate imaging studies indicative of the measurable target lesions every three chemotherapy cycles. Patients with disease regression or stabilisation received up to six chemotherapy cycles. Patients with PD were withdrawn from the study. The duration of response was measured from the first documentation of response to disease progression.

### Statistical analysis

Patients were evaluated for response on an intention-to-treat basis, and patients who received at least one cycle of treatment were evaluable for toxicity. Toxicity and DLT analyses were carried out regarding patients entering the phase I evaluation, and after the recommended level for phase II testing was defined, analysis of toxicity in the phase II part of the study was carried out separately. Response duration was measured from the day of its initial documentation until confirmed disease progression; TTP was calculated from study entry until evidence of PD; OS was measured from the day of entry until last follow-up or death. In the phase II part of the study, the 95% confidence intervals (CI) for RRs were calculated from the binomial distribution (Simon, 1986). Median duration of response, median TTP, and actuarial OS were estimated by the product-limit method of Kaplan–Meier (Kaplan and Meier, 1959). According to the two-stage design reported by Simon (1989) for phase II studies, with an expected RR of 50% and a worst RR of 30%, 28 patients would be required in the first step. If a minimum of seven responses were encountered, a total of 39 patients would be accrued (min–max design). Thereby, the probability of accepting a therapy with a real RR less than 30% and the risk of rejecting a treatment with a RR greater than 50% would be in both cases less than 10%.

## RESULTS

### Patient characteristics

A total of 56 patients were entered in the present phase I–II study of docetaxel–ifosfamide combination between March 1997 and November 2000. Patients were entered at five consecutive DLs in the phase I part of the study (Table 1) as follows: 21 were treated in phase I DLs (DL1: 3, DL2: 6, DL3: 3, DL4: 6, and DL5: 3) and the remaining 35 were treated at DL4, which was defined as the level for phase II testing. In total, 41 patients were treated at DL4 that was defined as the MTD (see below). Patients characteristics are demonstrated in Table 2

**Table 2:** Patient characteristics

**Characteristic**	**No.**	**%**
Total patients	56	100
		
*Age (years)*		
Median	54.5	
Range	32–72	
		
*Performance status (WHO)*		
0–1	48	86
2	8	14
		
*Menopausal status*		
Premenopausal	20	36
Postmenopausal	36	64
		
*Histology*		
Ductal	42	75
Lobular	5	9
Not specified	9	16
		
*Hormone receptors*		
ER−/PR−	13	23
ER+/PR−	10	18
ER+/PR+	20	36
ER−/PR+	7	12
Not done	6	11
		
*No. of prior regimens*		
1	30	53
2	24	43
3	2	4
		
*Type of chemotherapy*		
Adjuvant CAF	7	12.5
Neoadjuvant	7	12.5
Metastatic disease	24	43
Adjuvant+metastatic	18	32
		
*Anthracycline sensitivity*		
Sensitive	23	39
Refractory	33	61
		
*Metastatic sites*		
1	30	53
2	43	43
⩾3	2	4

. All patients were evaluable for toxicity (*n*=56) and 39 out of 41 patients entered at DL 4 were assessable for response in the phase II part of the study. The median age was 54.5 (range, 32–72) years, the median WHO performance status was 1 (range, 0–2); being 0–1 for 48 (86%) patients. In all, 33 (59%) patients were anthracycline-refractory and 23 (41%) were potentially anthracycline-sensitive according to the definitions. The median number of prior chemotherapy regimens including adjuvant and/or anthracycline-based treatment was 1 (range, 1–3). The median interval from the end of the last chemotherapy regimen was 6 (range, 1.5–45) months. All patients received at least one chemotherapy cycle and were therefore evaluable for toxicity, while 53 out of 56 patients received at least two chemotherapy cycles and were therefore evaluable for response. Dose-limiting toxicity was reached at DL5 with two out of three initial patients developing febrile neutropenia.

### Toxicities

#### Phase I

Five DLs were evaluable for toxicity in the phase I part of the current study. No DLTs were observed at DL1. At DL2, three out of six patients developed febrile neutropenia after the first cycle. The same DL was repeated with the addition of prophylactic G-CSF as DL3, and none of the three patients entered developed DLT. At DL4, one out of six patients entered developed febrile neutropenia. At DL5, two our of three initial patients developed febrile neutropenia and one of these developed sepsis and grade 3 diarrhoea managed successfully by broad-spectrum antibiotics and other supportive care measures; neither further accrual of patients was undertaken nor further dose escalation was attempted beyond DL5 according to our preset definitions. No other important haematologic or nonhaematologic grade 3/4 DLT was observed in phase I (Table 3

**Table 3:** Results of docetaxel–ifosfamide dose escalation in phase I

**DL**	**No. of patients**	**No. of treatment cycles**	**DLT**	**Type of toxicity (Grades 3 and 4)**
1	3	18	0/3	None
2	6	29	3/6	3 Gr4 FN
3	3	15	0/3	None; Gr4 neutropenia of < 7 days duration in one patient
4	6	32	1/6	1 Gr4 FN
5	3	16	2/3	2 Gr4 FN; (one of these with sepsis and Gr3 diarrhoea)

DL=dose level, DLT=dose-limiting toxicity (after first cycle), FN=febrile neutropenia.

).

#### Phase II

Haematologic and nonhaematologic toxicities encountered in the present study were evaluated in all the patients and cycles and are shown in Table 4

**Table 4:** Haematologic toxicities (NCI-CTC grade) (phase II part of the study)

	**NCI-CTC grade (% of patients, all cycles)**				
**Toxicity**	**0**	**1**	**2**	**3**	**4**
Leukopenia	11	4	4	46	35
Neutropenia	11	0	11	15	63
Thrombocytopenia	58	32	10	0	0
Anaemia	5	25	55	15	0
Febrile neutropenia	12%				

and Table 5

**Table 5:** Nonhaematologic toxicities (NCI-CTC grade) (phase II part of the study)

	**NCI-CTC grade (% of patients, all cycles)**				
**Toxicity**	**0**	**1**	**2**	**3**	**4**
Nausea and vomiting	54	27	14	5	0
Mucositis	37	37	22	0	0
Myalgia/arthralgia	61	24	15	0	—
					
*Neurologic*					
Peripheral	32	54	14	0	0
CNS	59	22	17	0	2
Infection	97.5	2.5	0	0	0
Diarrhoea	41	37	12	10	—
Hypersensitivity reactions	93	7	0	0	0
Alopecia	0	0	100	0	—
Skin/nail	41.5	41.5	17	0	0
Fluid retention	61	37	2	0	—
Asthenia/fatigue	27	41.5	22.5	10	—
Cardiac	100	0	0	0	0
Pulmonary	96	2	0	2	0
Renal	97.5	0	0	2.5	0
Haematuria	98	2	0	0	0

, respectively. In brief, grade 3/4 toxicities included neutropenia 32 out of 41 (78%)–with 26 out of 41 (63%) developing grade 4 neutropenia (⩽7 days) and five of these febrile neutropenia (12%). All were managed successfully with broad-spectrum antibiotics. One patient with extensive liver metastases but no pretreatment deterioration of liver function (according to the eligibility criteria) developed severe metabolic acidosis, uric acid, liver enzyme, and bilirubin elevation 16 h after the first dose of docetaxel and ifosfamide and died as a result of that complication 48 h later from multiorgan failure; her death could be attributed to either drug-related hepatic toxicity or acute tumour lysis syndrome, or a combination of all these factors. No other treatment-related deaths were observed. No grade 3 or 4 thrombocytopenia was observed. Anaemia was cumulative in nature and six out of 41 patients required packed red blood cell transfusions for grade 3 anaemia. Other nonhaematologic toxicities are shown in detail in Table 5. One patient developed grade 1 and one grade 3 pulmonary toxicity, after the fifth and fourth cycle of treatment, respectively. The latter patient developing grade 3 pulmonary toxicity with diffuse alveolar infiltrates and hypoxaemia improved rapidly after reinstitution of steroids, but further treatment was witheld.

### Compliance to treatment

In phase I part of the study, six patients who developed DLTs continued with 20% dose reduction (see Table 3 also).

In phase II (including all patients entered at DL4), a total of 190 treatment cycles were administered; median: 6 (range: 1–6), with a mean of 4.6 cycles per patient. In total, 16 patients did not complete the planned six cycles because of the following reasons: 12 patients (one after cycle 1, seven after cycle 2, and four after cycle 3) because of PD; one because of toxic death (liver failure) after cycle 1, and three after cycle 4 because of personal choice; two patients and grade 2 asthenia/fatigue; one patient. A total of 13 treatment cycles (7%) were delayed for 2–14 days (median: 7 days) for the following reasons: patient's own choice or logistic reasons of travelling from district areas; seven cycles, transfusion for Grade 2/3 anaemia; four cycles, neutropenia (with neutrophils <1500 *μ*l^−1^) on the day of treatment; two cycles.

### Dose-intensity analysis

The administered median dose intensities for each drug of the docetaxel/ifosfamide combination in the phase II part of the study were as follows: for docetaxel 30.75 mg m^−2^ week^−1^ (range: 24.2–33.3), and for ifosfamide 1.54 g m^−2^ week^−1^ (range: 1.24–1.67), that is, 92% (range: 72.6–100%) of the planned dose intensities for both drugs.

### Response to treatment and survival

In total, 53 patients were assessable for response when both phase I and II parts of the study (all DLs) were considered. Overall, five CRs and 24 PRs were recorded, for a 52% (95% CI: 39–65%) RR on intention to treat (see also Table 6

**Table 6:** Response to docetaxel–ifosfamide (all levels); *n*=56 patients

	**No. of assessable **	**No. of responses**				
**DL**	**patients**	**CR**	**PR**	**SD**	**PD**	**ORR (%)**
1	3	1	0	1	1	33
2	6	0	3	2	1	50
3	3	1	1	1	0	67
4 (phase II)	41	3	19	7	12	53.6
5	3	0	1	1	1	33
Total	56	5	24	12	15	52

ORR=overall response rate.

). When RRs were divided according to prior anthracycline sensitivity; 13 out of 23 (56.5%; 95% CI, 34.5–76.8%) of anthracycline-sensitive patients *vs* 16 out of 33 (48.5%; 95% CI, 30.8–66.5%) of anthracycline-resistant patients responded, and the difference did not reach significance. Overall, median duration of response was 7 (3–24) months, median TTP 6 (0.1–26) months, and median OS 12 (0.1–33) months. Median duration of response, median TTP, and median OS for anthracycline-sensitive patients were: 9 (3–24), 6.5 (0.2–26), and 13 (1–33) months, respectively, while for anthracycline-refractory patients these were: 6.5 (3–14+), 5 (0.1–16), and 12 (0.1–25+) months, respectively.

Clinical RRs, on an intention-to-treat basis, in phase II (MTD) were as follows: 22 out of 41 (53.6%; 95% CI, 38.3–68.9%) patients responded; three CRs, 19 PRs, seven SD and 12 PD (Table 6). Again, no difference in RRs was observed between anthracycline-sensitive and anthracycline-refractory patients. The patient who died as a result of toxicity and the patient with rapid progression, both after the first cycle were considered as having PD. The median response duration was 7 (3–24) months, median TTP 6.5 (0.1–26) months, and median OS 13 (0.1–33) months.

## DISCUSSION

The rationale for combining taxanes paclitaxel or docetaxel with ifosfamide derives from both *in vitro* data and theoretical assumptions based on the properties of each individual cytotoxic agent to mediate its cellular damage. Most *in vitro* data exist with paclitaxel. In brief, paclitaxel inhibits the energy-dependent enzymatic reactions, by disengaging activated intracellular phosphate (e.g. ATP and GTP), required for the repair of the DNA damage induced by alkylating agents (prevention of DNA strand separation and unwinding) (Reed *et al*, 1995). *In vitro* synergism has been demonstrated between paclitaxel and hydroperoxy-ifosfamide, an activated ifosfamide metabolite, against cisplatin-sensitive and -resistant Ovarian Carcinoma cell lines when paclitaxel preceded hydroperoxy-ifosfamide or during concurrent exposure (Liebmann *et al*, 1994; Klaassen *et al*, 1996). 

Based on these preclinical *in vitro* experimental data, we believe that the sequence and infusion times regarding docetaxel and ifosfamide, as applied in the present study, might lead to potential *in vivo* synergism between these two drugs (Kearns *et al*, 1995). Moreover, our prior experience with paclitaxel–ifosfamide–cisplatin (Kosmas *et al*, 2000a,2000b,^2001^) or docetaxel–ifosfamide–cisplatin (Kosmas *et al*, 2002) combinations has demonstrated their feasibility in phase I and phase II studies in advanced solid tumours and lung cancer in particular.

If the above considerations regarding sequence-dependent interactions for optimal drug scheduling are important in order to maximise efficacy, of equal importance are the effects of drug sequencing related to bone marrow toxicity. Data from phase I clinical studies of the paclitaxel/cyclophosphamide combination employing different schedules of drug administration demonstrated variable haematologic toxicity. The highest degree of haematologic toxicity was encountered when paclitaxel was administered by 24-h or 72-h continuous infusion with high doses of cyclophosphamide (Kennedy *et al*, 1996; Tolcher *et al*, 1996). However, when paclitaxel, given by 3-h infusion, was followed by cyclophosphamide, bone marrow toxicity was of much less severity (Pagani *et al*, 1997). In contrast, with the docetaxel–ifosfamide combination, the schedule of administering the taxane first led to less haematologic toxicity and a higher MTD than did the reverse drug sequence (Pronk *et al*, 1998). It is therefore realistic to consider that the sequence of administration of docetaxel followed by ifosfamide could account for the tolerable haematologic toxicity, that is, neutropenia and thrombocytopenia, encountered in our study up to high individual drug doses that were achieved at DL4. Moreover, as grade 4 neutropenia and febrile neutropenia represented the only significant toxicities in our study, the 12% incidence of the latter appears rather low and compares favourably to that of single agent docetaxel at 100 mg m^−2^ with G-CSF support. The phase I study of Pronk *et al* (1998) that has evaluated the feasibility of the docetaxel–ifosfamide combination without G-CSF in pretreated patients with a variety of advanced solid tumours determined the DLT of the combination being mainly neutropenia at the following doses; docetaxel 85 mg m^−2^ on day 1 followed by ifosfamide 5 g m^−2^ administered as 24-h infusion, and the recommended phase II doses were docetaxel 75 mg m^−2^ +ifosfamide 5 g m^−2^ (Pronk *et al*, 1998). A subsequent pharmacokinetic analysis of the regimen by the same investigators found that the sequence of drug administration did not affect the clearance and the area under the curve (AUC) of docetaxel. However, there was a decrease in the AUC of ifosfamide in the schedule of docetaxel→ifosfamide compared with the reverse sequence (Schrijvers *et al*, 2000). It is also possible that ifosfamide might yield a decreased AUC when administered by 24-h continuous infusion compared to short 1–2-h infusions fractionated over 2 or more days (Cerny *et al*, 1999). 

Ifosfamide combinations in advanced anthracycline-pretreated breast cancer have been applied in recent years. Combination of ifosfamide with vinorelbine demonstrated an RR of 56% in a group of patients with no or minimally pretreated metastatic breast cancer (Leone *et al*, 1996). The combination of a fixed dose of doxorubicin 20 mg m^−2^ × 3 days with escalating doses of ifosfamide (1.2–2.75 g m^−2^ day^−1^ × 5 days) with G-CSF support in a phase I study focusing in stage IV chemotherapy-naïve breast cancer has yielded the feasibility of a quite high dose of ifosfamide 12.5 g m^−2^ (total) with an RR of 83% of which 33% were CRs (Bitran *et al*, 1995). 

Moreover, ifosfamide has been combined with paclitaxel in a phase I study in patients with advanced heavily pretreated predominantly breast and ovarian malignancies including 13 patients with advanced anthracycline-pretreated breast cancer (Bunnell *et al*, 1998). While the MTD reached for paclitaxel was 190 mg m^−2^ by 24-h infusion and for ifosfamide 3.0 g m^−2^ day^−1^ for 3 days (total dose: 9.0 g m^−2^), no major toxicities were en-countered with this quite high dose of ifosfamide administered by short noncontinuous daily infusion, while RRs in breast cancer patients were almost 62% with 31% CRs in this study (Bunnell *et al*, 1998). 

Another phase II study evaluating docetaxel and ifosfamide in women with heavily pretreated anthracycline- and hormone-refractory breast cancer led to early disappointment in view of no responses seen in the first 10 patients entered (Lorusso *et al*, 2000). 

In the aforementioned phase I–II study of Pagani *et al* (1997) evaluating the paclitaxel/cyclophosphamide doublet, a dose–response effect was suggested for pretreated patients, with a lower RR reported for those receiving <1500 mg m^−2^ of cyclophosphamide (Pagani *et al*, 1997). As equivalent cytotoxic alkylator doses of ifosfamide are anticipated at 4.5–6.0 mg m^−2^, the recommended phase II dose derived from our study regarding ifosfamide might represent an optimal alternative to cyclophosphamide with less haematologic toxicity.

The value of ifosfamide in relapsed anthracycline-pretreated advanced breast cancer as a single agent or in combination, despite promising phase II results, cannot currently be defined in the absence of randomised data. As the combination of docetaxel+ifosfamide in the phase II part of the present study yielded a 54% RR, it can be argued that similar results might have been obtained with single-agent docetaxel. However, the almost 55% RRs obtained with single-agent docetaxel in the early phase II studies (Ravdin *et al*, 1995; Valero *et al*, 1995) should be regarded as preliminary, since these have been based in small numbers of patients and subsequent randomised studies of single-agent docetaxel *vs* MMC+VLB or *vs* MF in anthracycline-refractory or heavily pretreated patients yielded RRs of 30 and 42%, respectively, for docetaxel (Nabholtz *et al*, 1999; Sjostrom *et al*, 1999). Moreover, it should be emphasised that ours was a dose-finding/preliminary efficacy phase I–II study, with a 12% incidence of febrile neutropenia, which is still of concern in the setting of palliative chemotherapy.

Therefore, randomised phase III studies of single-agent docetaxel at 100 mg m^−2^ *vs* the combination of docetaxel+ifosfamide, as defined in the present study, might address the value of adding ifosfamide to docetaxel, as well as the issue of cost effectiveness of such a combination, since it is well appreciated that ifosfamide administration is rather cumbersome, even in the outpatient setting, expensive and requires multiple admissions (over 2–5 days) for each cycle. However, it should be kept in mind that until recently combination chemotherapy has never shown any clear survival benefit over single agents in randomised trials, while instead inducing more toxicity in some studies. Recent results with the docetaxel+oral capecitabine (Xeloda®) combination *vs* single-agent docetaxel in a phase III randomised North American trial have yielded the superiority and convenience of the combination in anthracycline pretreated breast cancer (O’Shaughnessy *et al*, 2002), thus providing a docetaxel combination of more clinical interest. At present, our results can be viewed as having defined the MTD, the recommended phase II doses and RR of the docetaxel+ifosfamide combination, which warrant further randomised phase III comparisons to docetaxel monotherapy or other active combinations of docetaxel with newer agents, such as gemcitabine, vinorelbine, or capecitabine.
